# Improving of 100Cr6 Steel Corrosion and Wear Properties in Simulated Sea Water Environment by Tungsten-Doped DLC Coating

**DOI:** 10.3390/ma16124334

**Published:** 2023-06-12

**Authors:** Martin Vicen, Daniel Kajánek, Libor Trško, Otakar Bokůvka, Martin Buchtík, Zuzana Florková, Martin Frkáň

**Affiliations:** 1 Department of Materials Engineering, Faculty of Mechanical Engineering, University of Zilina, Univerzitná 8215/1, 010 26 Zilina, Slovakia; martin.vicen@fstroj.uniza.sk (M.V.); otakar.bokuvka@fstroj.uniza.sk (O.B.); 2 Research Centre, University of Zilina, Univerzitná 8215/1, 010 26 Zilina, Slovakia; daniel.kajanek@uniza.sk (D.K.); zuzana.florkova@uniza.sk (Z.F.); 3 Materials Research Centre, Faculty of Chemistry, Brno University of Technology, Purkyňova 464/118, 61200 Brno, Czech Republic; buchtik@fch.vut.cz; 4 Staton, s. r. o., Sadová 1148, 038 53 Turany, Slovakia; frkan@staton.sk

**Keywords:** DLC coating, W-DLC coating, wear, tribology, corrosion resistance

## Abstract

A progressive type of tungsten-doped DLC coating was applied to a quenched and tempered 100Cr6 steel with the aim to improve the wear and corrosion properties in simulated seawater conditions and to compare the performance to conventional DLC coating. Tungsten doping caused a shift of the corrosion potential (E_corr_) to a lower negative value of −172 mV, while the conventional DLC exhibited an E_corr_ of −477 mV. In dry conditions, the W-DLC coefficient of friction is slightly higher than that of the conventional DLC (0.187 for the W-DLC vs. 0.137 for the DLC), but in cases of a saltwater environment, this difference becomes almost negligible (0.105 for the W-DLC vs. 0.076 for the DLC). The conventional DLC coating also started to show marks of deterioration when exposed to a combination of wear in a corrosive environment, while the W-DLC layer still maintained its integrity.

## 1. Introduction

Roller bearings are one of the most important industrial components with a large variety of challenging environmental applications [1]. In nearshore and offshore applications, the bearings are required to resist increased air salinity, which, in combination with moisture, forms a NaCl water solution—which is highly corrosive for all ferritic materials. Even though roller bearings are highly lubricated, besides the decreased friction, they also provide a good protective boundary between the bare metal and the surrounding environment (the lubricants are often forced out of the zones with high contact pressures). In this case, only a very thin film is left on the surface with limited corrosion protection due to possible element diffusion through the polymer layer. Polymer lubricants also have the tendency to dissolute small amounts of water, which, again, creates a corrosive environment.

Surface coatings are typically the first choice for corrosion resistance improvements [2], however, their application on rolling bearings is really challenging, due to high contact stresses and rolling wear, which quickly wears down most of the conventional coatings. Currently, one of the most durable and wear-resistant coatings are manufactured using the physical vapor deposition (PVD) process. Diamond-like carbon (DLC) coatings appear promising for bearing application due to their high corrosion and wear resistance and their thickness lies typically between 1 and 2 μm, thus, not affecting the bearing geometry significantly [2,3,4,5].

Milewski et al. [6] performed a wear analysis on the roller bearings of belt conveyors coated with plasma-assisted DLC after 1.5 years of operation. The coated bearings were able to be in service three times longer than the non-coated bearings. Vanhausel et al. [7] tested the application of highly hydrogenated DLC coatings on AISI 52100 steel substrates for ball bearings used in space applications. Ball-on-disc tests have shown that the tribological performance strongly depends on the deposition conditions and the tribotest environment. However, when proper process parameters were applied, the coatings were able to provide excellent tribological performance in a vacuum combining a lifetime of 100,000 cycles with a very low coefficient of friction (0.007 in steady-state regime).

Even when most of the published results show very promising results, proper coating deposition is not trivial, and intense process optimization is required. The final performance of PVD coatings is in general sensitive to the type of substrate material, process temperature [8], surface roughness [9], coating thickness [10,11], surface cleanliness, and so on. Despite the sensitivity of the process, PVD and especially DLC coatings were already well adapted in industries such as military equipment [12].

In addition to the excellent wear properties, the DLC coatings also provide excellent corrosion protection for the majority of metallic materials in various environments. The DLC coating on the SUS316 steel and Ni-Cr-Mo alloy (Hastelloy) showed that it provides superior corrosion resistance when compared to the substrate materials [13]. The authors of the work [14] proved evidence of high DLC coating stability in an acid solution and in another work [15], the authors showed the improvement of the SU 304 stainless steel’s corrosion resistance in a 3.5% NaCl solution. The presence of a DLC coating improved the corrosion resistance 10.9 times compared to the SU 304 austenitic stainless steel base material. When the coating was exposed to a simulated marine atmosphere, the DLC-coated specimens were proven to be more resistant than the galvanized coatings [16]. Wang et al. studied the DLC tribological performance, showing that the coating maintained a low friction coefficient even in a corrosive environment while still providing high corrosion protection [17].

The number of available works focused on the tribology behavior of tungsten-doped DLC coatings is still very limited. A systematic study was carried out to assess the influence of the tungsten content on the tribological properties of the tungsten-doped diamond-like carbon (W-DLC) coatings, lubricated using molybdenum dithiocarbamate, was performed by Yue et al. [18]. The results have shown a constant coefficient of friction (COF) with a value of 0.06 with no influence on the tungsten content. The tribological behavior of the W-DLC coating under oil lubrication was studied by Križan et al. [19]. Results have shown that in the majority of the tested oils, the COF of the coating was lower when compared to the bare steel substrate. Another important work was performed by Chen et al. [20] where the mechanical and tribological properties of the tungsten-gradually-doped DLC films, with a functionally graded interlayer, prepared using a hybrid technique of vacuum cathodic, arc/magnetron, sputtering/ion beam deposition, were analyzed. In the films, a functionally graded interlayer, with an optimized sequence of Cr/CrN/CrNC/CrC/WC, was first deposited, and then a diamond-like carbon layer, doped with a gradually decreasing content of W, was coated. The optimum wear performance (COF = 0.19) was achieved for the W-gradually-doped DLC film with a graded W concentration from 52.5% to 17.8%. It must be noted that the COFs of all the tested W-DCL coatings in dry conditions were higher than those of a conventional DLC layer.

On the contrary, the DLC-type coatings are not expected to have a significant influence on surface pitting, which is the most often and severe failure mechanism of roller bearings. Pitting damage occurs due to high shear stresses under the surface caused by contact loading [1]. Since the position of the maximal shear stresses is typically under the coating, deeper in the surface layers of the substrate, the effect of the coating can be negligible. In the case of coating spalling due to poor adhesion, the loose particles can result in severe surface damage to the rolling surfaces, causing premature bearing failure. Another negative aspect of DLC coatings, which prevents their wide application, is a notable price increase on the final product. This is why they are applied only in situations where the positive effects will result in higher cost savings in maintenance and bearing replacement, than in the cost of the coating.

The objective of this study was to experimentally evaluate the tribological and corrosion properties of a progressive tungsten-doped DLC coating (W-DLC), which was modified to have higher hardness and better layer compactness when compared to conventional DLC coatings. The coating was applied to quenched and tempered 100Cr6 steel to simulate a potential application on roller bearings in an aggressive environment.

## 2. Experimental Material and Coating Process

For experimental analysis, plates of 100Cr6 steel, with a length of 80 mm, width of 30 mm, and thickness of 5 mm, were used. The chemical composition of the experimental material was analyzed using optical emission spectroscopy (OES) on a SPECTROMAXx device (SPECTRO Analytical Instruments, Kleve, Germany). The 100Cr6 steel plates were hardened per the following quenching and tempering (QT) process: austenitization at 840 °C ± 5 °C for 20 min, cooled in oil to 20 °C ± 2 °C, tempering at 160 °C ± 5 °C for 90 min, and finally cooled in open air. After the heat treatment, the microstructure was studied with the use of standard light microscopy and scanning electron microscopy (SEM). The hardness of the experimental material, after heat treatment, was determined using the Rockwell hardness test (HRC) on an RR-10/AQ tester (Aquastyl, Povazska Bystrica, Slovakia). A minimum of five indentations were performed on each plate. Prior to coating deposition, the faces of the plates were first mechanically polished to a roughness of Ra = 0.039 µm and Rz = 0.397 µm and ionically cleaned to remove impurities from their surface before the coating deposition. All processes, including the coating depositions, were performed at temperatures lower than the tempering temperature to avoid secondary tempering of the substrate, which would result in a further decrease in hardness.

The conventional DLC deposition was performed with the use of the OCTOMAG M250 coating system unit, which consists of six unbalanced magnetrons cathodes with diameters of four inches (UBDCMS), connected to DC power sources, TruPlasma DC 3005 (Trumpf Hüttinger, Freiburg im Breisgau, Germany) and a TruPlasma Highpulse Series 4000 (G2) (Trumpf Hüttinger, Germany). Four of the magnetrons were equipped with Cr targets and the remaining two magnetrons with C targets, all with a purity of 99.5% (Testbourne, Basingstoke, UK). Two of the C magnetrons were connected to a high-power impulse magnetron sputtering (HiPIMS) power supply, TruPlasma Highpulse Series 4000 (G2) (Trumpf Hüttinger, Germany). The remaining four Cr magnetrons were supplied by a DC power supply, TruPlasma DC 3005 (Trumpf Hüttinger, Germany). The coating chamber was connected through a cold trap to a vacuum pumping system consisting of rotary and diffusion pumps. The system was pumped down to a base pressure below 1 × 10^−3^ Pa before each deposition. The 100Cr6 substrates were mounted onto a rotatable substrate holder which was connected to a high-voltage source, TruPlasma DC 4020 (G2) (Trumpf Hüttinger, Germany). Argon, acetylene (C_2_H_2_), neon, and nitrogen were used as sputtering and reactive gases, respectively. The substrate was heated to 100 °C for 1 h and then the surface was pretreated using an ion source in an Ar atmosphere at 0.1 Pa. Firstly, the Cr adhesion interlayer was deposited using DC magnetron sputtering (DCMS) of two Cr targets in the Ar atmosphere at 0.3 Pa. Finally, the DLC coating was deposited using reactive high-power impulse magnetron sputtering (HiPIMS) from the two C targets in a mixture of C_2_H_2_, Ne, and N gases at 0.3 Pa. The substrate selective bias was at the potential of −500 V during the deposition [21]. The total process time including the pre-heating was 5 h.

The W-DLC coating was deposited using DCMS. For the study, a proprietary coating system unit was used that consists of six unbalanced DC magnetrons with diameters of four inches (UBDCMS) connected to a DC power source, TruPlasma DC 3005 (Trumpf Hüttinger, Germany). Four of the magnetrons were equipped with composite ceramic tungsten carbide targets and the remaining two magnetrons with Cr targets, all purities of 99.5% (Testbourne, UK). One of the Cr magnetrons could be alternatively connected to a HiPIMS power source, TruPlasma HighPulse 4002 (Trumpf Hüttinger, Germany). The coating chamber was connected through a cold trap to a vacuum pumping system consisting of rotary and diffusion pumps. The system was pumped down to a base pressure below 1 × 10^−3^ Pa before the deposition. The plasma composition and the stability of the pretreatment and deposition processes were monitored in situ using an optical emission spectroscope (OES) HR4000CG-UV–NIR (Ocean Optics, Orlando, FL, USA). The spectra were recorded in the position of the substrates. The obtained spectra were analyzed using the atomic spectra database provided by the National Institute of Standards and Technology. The 100Cr6 substrates were mounted onto a rotatable substrate holder which was connected to a high-voltage source, TruPlasma Bias 3018 (Trumpf Hüttinger, Germany). The holder was rotated at a speed of 2.5 rpm during the substrate’s pretreatment and the tungsten carbide coating was deposited. Argon and acetylene (C_2_H_2_) were used as sputtering and reactive gas, respectively. The substrate was heated to 100 °C for 1 h. The substrate selective bias was at the potential value of −600 V during the deposition [21]. The total process time, including the pre-heating, was 5 h.

The thickness of the coatings was measured using a Calotest (CSM Instruments, Switzerland) [22].

The dry and wet (3.5% NaCl solution—sea water simulation) coefficient of friction of the DLC and W-DLC coatings were experimentally determined on a linear sliding tribometer using the ball-on-flat test method per ASTM G-133. After performing 5000 cycles of rectilinear reciprocating motion of the WC ball with 3 mm in diameter over the surface at the sliding speed of 0.1 m⸱s^−1^, the test was terminated (the test duration was approximately 42 min). During the tribological test, the sliding WC ball was loaded using a normal force of F_N_ = 12 N. The experimental data, obtained by linear tribometer, were recorded and processed in the Signal Express software (v. 2015). The data were smoothened using an IIR filter of the second order with the low-pass cutoff at 16 Hz.

Coating adhesion properties, after the deposition, were evaluated using a scratch test, which is based on a load vs. displacement measurement and provides information on cohesive and adhesive failures, adhesion strength, and cracking. This test is essential to verify the deposition process. For example, insufficient surface sputtering (remaining surface contamination) results in poor coating adhesion and the high thickness of the coating causes the presence of high residual stresses, resulting in premature cracking of the coating under mechanical loading [10]. The scratch tests were performed on a Bruker UMT device (Bruker, Billerica, USA), with a Rockwell standard diamond tip at the maximum load of F_N_ = 60 N with a length of 6 mm (three tests were performed on each coating). The adhesive-cohesive properties were evaluated from the morphology of the scratches [6]. Hardness (H_IT_) and elastic modulus (E_IT_) measurements were carried out on the base material and the DLC and W-DLC coatings, via nano-indentation, were performed on an Anton Paar NHT device (Anton Paar, Graz, Austria) with a standard Berkovich diamond tip. An indentation matrix with a size of 4 × 4 was used for the evaluation of each coating. The maximum load for each measurement was chosen so that the maximum penetration depth in the coatings did not exceed 10% of its expected thickness value [22].

**Figure 1:**
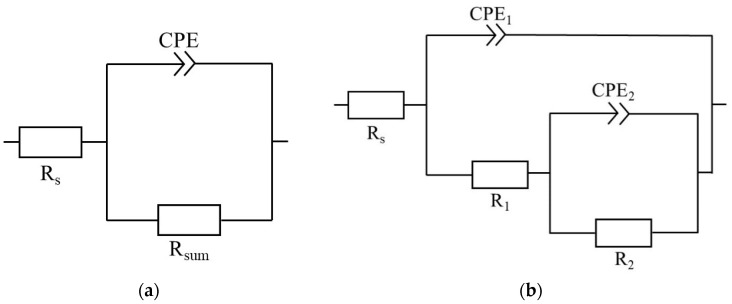
Equivalent circuits used for analysis of Nyquist diagrams with one capacitance loop (**a**) and two capacitance loops (**b**).

The corrosion resistance of plates, with the DLC and W-DLC coatings deposited on the base material, was evaluated using the potentiodynamic (PD) tests and electrochemical impedance spectroscopy (EIS) in a 3.5% NaCl solution at laboratory temperature after 1 h of exposure. A standard three-electrode cell system was used to connect to a laboratory potentiostat, Biologic SP300 (Biologic, Seyssinet-Pariset, France). Specimens were connected as working electrodes (Pt net as a counter electrode and saturated calomel electrode (SCE) as a reference electrode). The frequency during EIS measurements ranged from 100 kHz to 10 MHz, with a signal amplitude of 10 mV, and a mean value identical to the open circuit potential (OCP) [23]. Obtained data, in the form of Nyquist plots, were analyzed using corresponding equivalent circuits in Figure 1, where R_s_ represents solution resistance, R_1_ and R_2_ belong to resistances of corresponding semicircles in diagrams, and R_sum_ represents the sum of the partial resistances. The CPE components are called the constant phase elements and describe the heterogeneity of the electrode surface. More information about the circuit elements is presented in [24]. The PD tests were carried out in a range of potentials from −200 to +500 mV vs. the OCP. The potential was changed to a rate of 1 mV⸱s^−1^ [24]. The resulting values of the corrosion potential (E_corr_) and corrosion current density (i_corr_) were obtained using the Tafel analysis of the corresponding PD curves.

## 3. Results and Discussion

**Figure 2:**
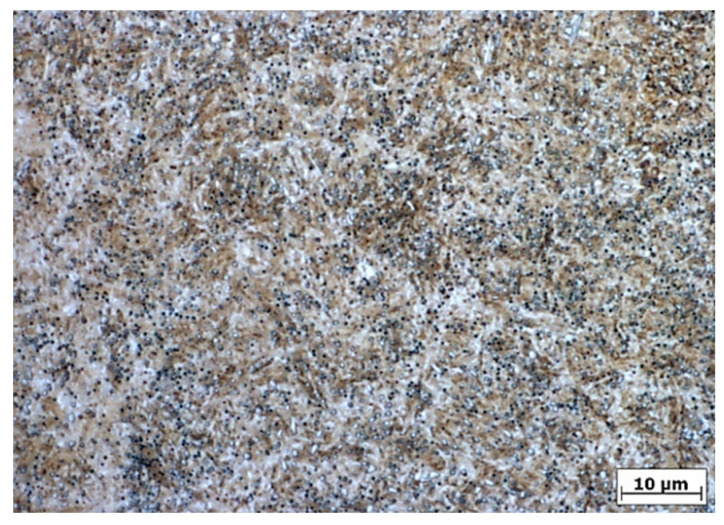
Microstructure of heat-treated base material, etched in 3% Nital, optical microscopy.

**Figure 3:**
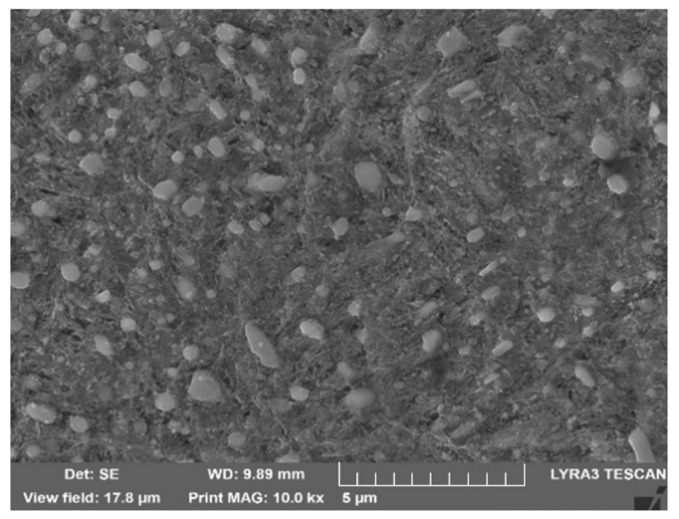
Microstructure of specimens heat treated base material, 3% Nital, SEM.

The chemical composition of the experimental material is given in Table 1. The microstructure, after quenching and tempering (Figure 2 and Figure 3), consisted of low-temperature tempered martensite and evenly distributed carbides. The hardness after the QT process reached a value of 62 HRC, which corresponds to the typical hardness requirement of roller bearings [1]. The retained austenite content, evaluated using X-ray diffraction (iXRD, Proto Manufacturing, Ontario, Canada), was less than 5% (austenite content is under the device resolution which is calibrated for min. austenite content of 5%).

**Figure 4:**
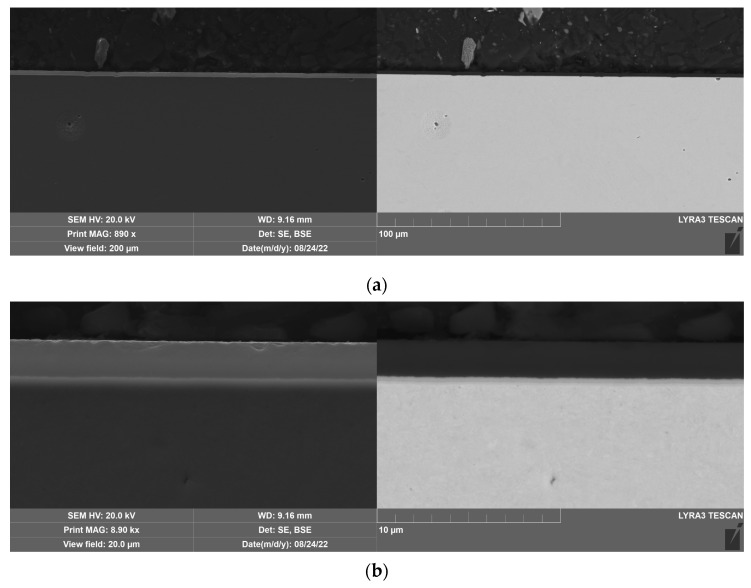
DLC coating deposited on the base material, unetched (**a**) general view, (**b**) detail.

**Figure 5:**
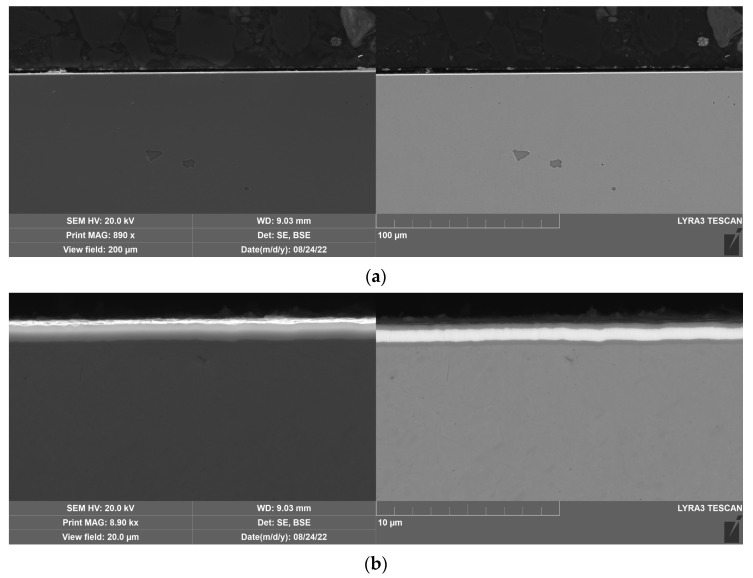
W-DLC coating deposited on the base material, unetched (**a**) general view, (**b**) detail.

The DLC and W-DLC coatings deposited on the base material observed on the transversal section through the coatings are shown in Figure 4 and Figure 5. The thickness (without the Cr interlayer) of the DLC coating was 2.77 ± 0.2 μm, while in the case of W-DLC coating, it was 1.15 ± 0.1 μm. No significant coating discontinuities or defects were observed along the whole length of the analyzed section.

**Figure 6:**
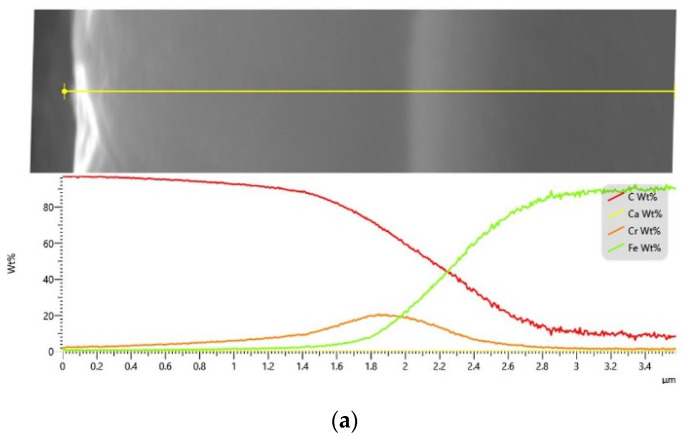

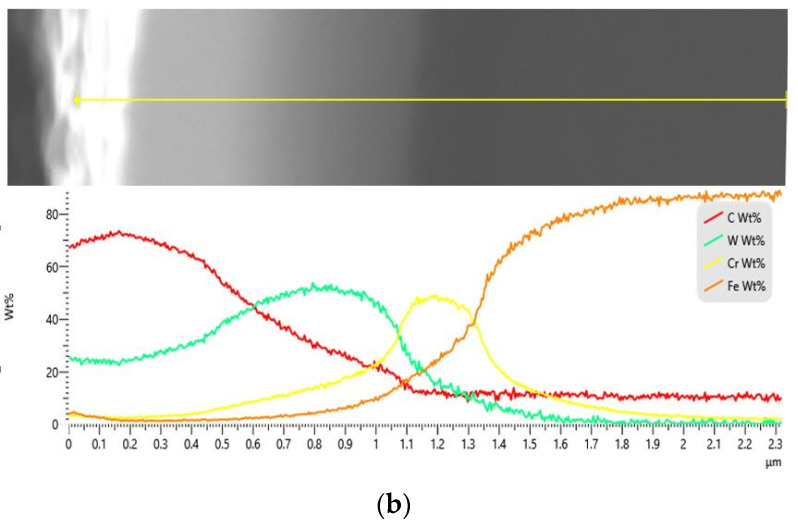
EDX analysis of the coatings, (**a**) DLC, (**b**) W-DLC.

The energy dispersive X-ray (EDX) analysis of both coatings (Figure 6) confirmed the expected presence of tungsten in the W-DLC coating when compared to the conventional DLC. The content of tungsten is not uniform through the thickness of the coating but starts at a value of 25 wt. % and increases to a maximal value of 50 wt. %, while the carbon content decreases. Additionally, the chromium interlayer used for better coating adhesion is visible and its thickness is approximately 1 µm for both coating types. The presence of regions with mixed element compositions instead of sharp boundaries indicates the diffusion character of the bonding, which results in good surface adhesion and a low risk of layer delamination.

The conventional DLC and W-DLC coatings scratch test was performed with increasing loading with a maximal value of F_N_ = 60 N. In the case of DLC coating, the first cracks appeared along the edges of the scratch at F_N_ = 29 N and the failure of the coating occurred at 45 N. First, the cracks of the W-DLC coating were observed at F_N_ = 22 N and the coating failed at F_N_ = 46 N. The scratch test results meet the expected values published in [2,5,10], which is the main indication of the proper deposition process and high quality of the coating.

The hardness measurement, via nanoindentation, of the base material (in QT condition), conventional DLC, and W-DLC coatings are shown in Table 2. Due to the large parameter variability of the coating process, it is possible to achieve a H_IT_ hardness between 10 GPa and 40 GPa and a modulus of elasticity (E_IT_) between 120 GPa and E_IT_ = 335 GPa [6,9]. In this case, the coatings were intentionally manufactured with a hardness on the lower side of the interval to keep it similar to the hardness of the substrate (Table 2) and to avoid the so-called “eggshell effect”. The “eggshell effect” occurs when the hardness of the coating is significantly higher than that of the substrate, thus, the substrate does not provide sufficient backing, and due to the elastic deformation of the substrate, the coating starts to crack. This also applies to the modulus of elasticity (Table 2) where the coating modulus is lower than that of the 100Cr6 substrate—the substrate is stiffer than the coating. Application of coatings with a higher hardness typically requires nitriding of the substrate steel [4].

**Figure 7:**
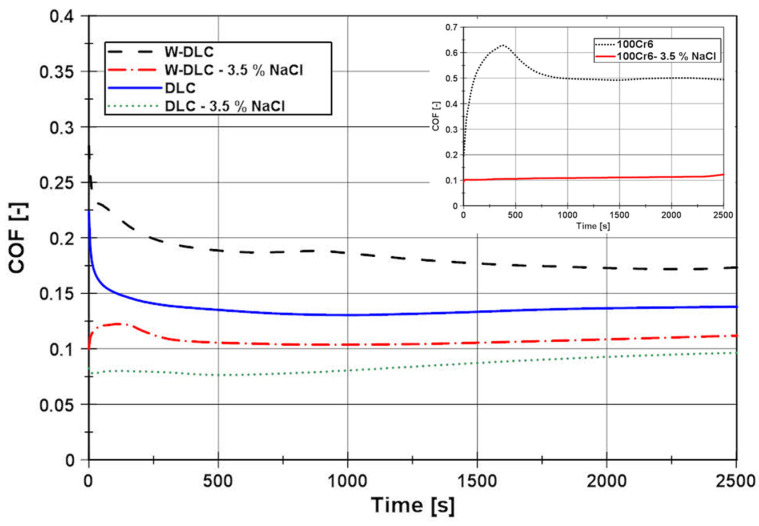
Comparison of the COF of the tribological pair, base material vs. DLC and W-DLC coatings, the dry and wet friction at F_N_ = 12 N load.

The measured results of the COF of the tested tribological pair in dry and wet conditions (3.5% NaCl solution), for the DLC and W-DLC coatings (base material is added for reference), are shown in Figure 7. The COF curves (Figure 7) can be divided into two regions. The first region is from the start until approx. 500 s, where the COF is decreasing. This is caused due to the wear of the roughness peaks in the first cycles as the initial roughness is decreasing to so-called “operating roughness”. After reaching the operating roughness, the curve continues in the second stage, where the COF is characterized by a lower value and more stable trend.

**Figure 8:**
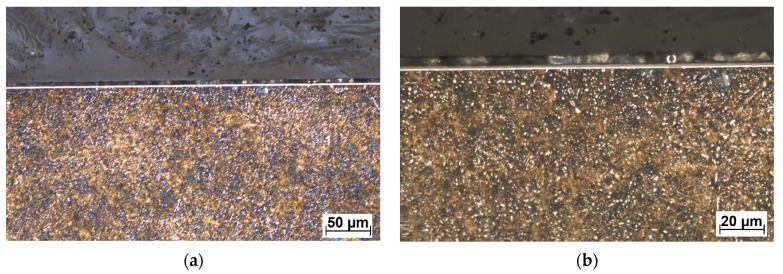
Cross-sectional metallography view of the dry friction wear trace of the conventional DLC coating (**a**), in detail (**b**), etched in 3% Nital.

**Figure 9:**
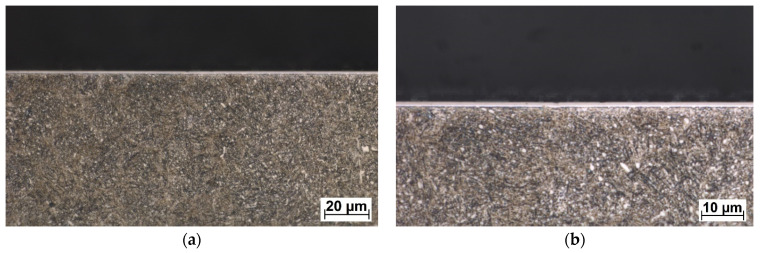
Cross-sectional metallography view of the dry friction wear trace of the W-DLC coating (**a**), in detail (**b**), etched in 3% Nital.

**Figure 10:**
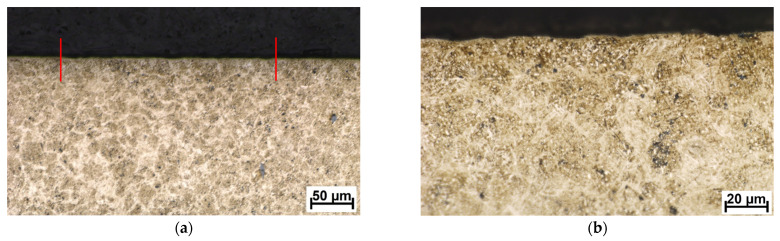
Cross-sectional metallography view of the dry friction wear trace of the substrate 100Cr6 steel (**a**), in detail (**b**), etched in 3% Nital. The width of the wear trace is designated by red lines.

According to the COF curves in Figure 7, in dry conditions, the COF, after 500 s of testing, stabilized at a value of ≈0.137 for the conventional DLC, and in the case of W-DLC, the COF was slightly higher and reached a value of ≈ 0.181. Even though the general hardness of the W-DLC coating is higher, the first stage of the COF curve (decreasing of roughness) is approx. the same for both coatings. This is caused by the fact that the contact pressures between the roughness peaks and the test ball are so high, that these are quickly worn down, thus, the relatively small increase in hardness of the W-DLC coating does not have a significant effect in the first stage of wear. In the second stage of wear, the COF trend of the conventional DLC and the W-DLS can be considered stable, since the changes in the value in this cycle range are insignificant. After performing the full 5000 cycles, no coating wear was visible neither on the conventional DLC (Figure 8), nor on the W-DLC coating (Figure 9). Both values are significantly lower than those of the base material with a COF of ≈0.5. For comparison, the cross-sectional view of a wear trace after performing an identical dry tribology test on the substrate 100Cr6 material is provided in Figure 10.

**Figure 11:**
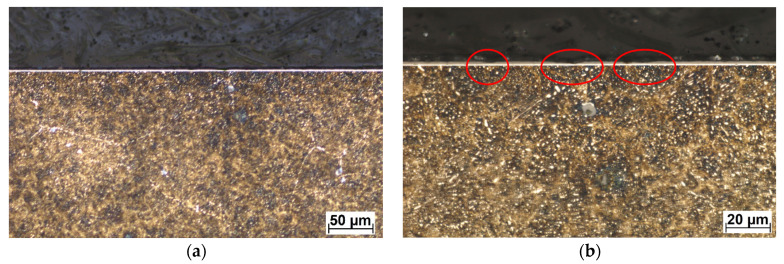
Cross-sectional metallography view of the wet friction wear trace of the conventional DLC (**a**), in detail (**b**), etched in 3% Nital. Coating damage is marked by red ellipses.

**Figure 12:**
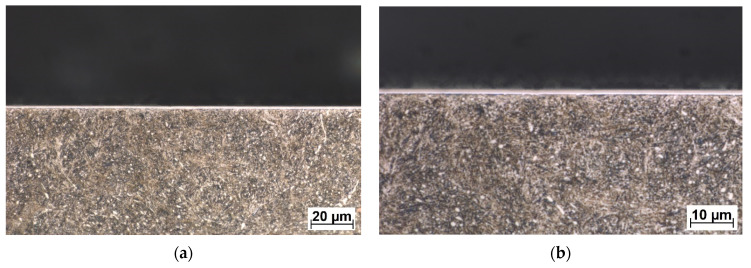
Cross-sectional metallography view of the wet friction wear trace of the W-DLC coating (**a**), in detail (**b**), etched in 3% Nital.

In wet conditions, the COF values have decreased when compared to the test in dry conditions. In the case of the conventional DLC, the average COF, after 500 s of testing, was ≈ 0.076, and in the case of the W-DLC, the COF was ≈0.105. Both COF curves have a very short first stage, which corresponds to a minimal surface roughness change since the contact area is lubricated by a 3.5% NaCl water solution. However, the conventional DLC COF at the end of the test (2500 s = 5000 cycles) reached a value of COF ≈0.1 (difference ΔCOF = 0.024). In the case of the W-DLC, the COF at the end of the test was 1.108 (difference ΔCOF = 0.003). The cross-sectional wear trace showed locations where the initiating surface damage was visible in the conventional DLC coating (Figure 11). In the case of the W-DLC coating, no damage was observed (Figure 12). The measured values are similar to the COF of the base material which reaches a value of ≈0.1, but at the end of the test (Figure 7), the COF is starting to increase, suggesting the initiation of severe wear damage (Figure 10).

**Figure 13:**
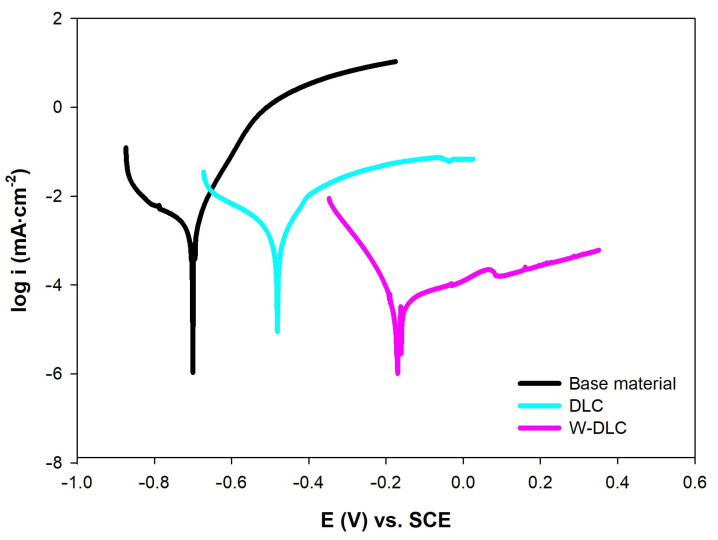
Potentiodynamic polarization curves of base material and coatings measured in 3.5% NaCl.

To analyze the corrosion performance of the coatings, electrochemical measurements, represented by the PD tests and EIS, were performed in a 3.5% NaCl solution. Results of the PD tests, presented in Figure 13 and Table 3, show that deposition of both coatings led to a shift in corrosion potential (E_corr_) towards the positive values with the W-DLC reaching even more positive E_corr_ compared to the DLC. These results suggest that the creation of both coatings led to the improved thermodynamic corrosion stability of the base material. The results are comparable with the work in [25], where the W-DLC coating dominated over the bare DLC coating in the artificial seawater, in terms of corrosion thermodynamics. The situation is similar in the case of corrosion kinetics, which is related to corrosion current density (i_corr_). It is obvious that, again, the coatings suppressed the corrosion process through the lower i_corr_ values. Deposition of the W-DLC provided the highest corrosion resistance, most likely due to the presence of tungsten within the coating.

**Figure 14:**
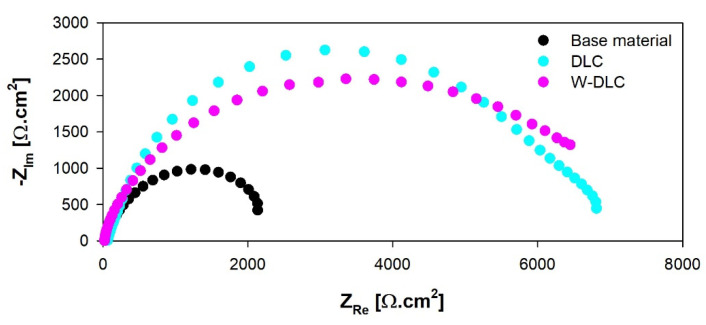
Nyquist diagrams of base material and coatings measured in 3.5% NaCl.

The results of the equivalent circuit analysis of the measured Nyquist plots, for all the tested series of specimens, are shown in Figure 14 and Table 4. In the considered case, the most important parameter, from the corrosion point of view, is given by the R_sum_ values, which represent the total resistance of the surface—meaning the higher the values are, the better corrosion resistance is obtained. Regarding this, both coatings improved the corrosion resistance of the examined steel to a considerable extent, while the W-DLC showed a slightly higher corrosion performance compared to the DLC coating. These findings are in good agreement with the results of the performed PD tests. As can be seen, the n values for the specimens varied from 0.8 to 0.9, which means that the capacitor had to be replaced by the constant phase element (CPE) for the proper analysis of the measured EIS spectra, suggesting that the behavior of the specimens slightly deviates from the ideal capacitor (n = 1). Additionally, according to the work in [24], the electrochemically active area is in direct relation to the value of CPE. When taking this into account, it can be stated that both coatings are represented by a lower electrochemically active area compared to the base material, which can be regarded as an additional beneficial contribution to the overall corrosion performance of the coated steel [26,27]. Generally, both corrosion techniques determined improved corrosion protection of the base material in the 3.5% NaCl, which can be biased slightly further when using the W-DLC coating. The improved barrier effect is therefore determined mainly by the beneficial presence of tungsten within the coating since the thickness of the W-DLC is lower compared to the thickness of the DLC coating. These findings are in agreement with the results presented in [21], meaning that the addition of W led to the higher compactness of the DLC coating.

The improvement of corrosion resistance by the DLC coating was expected since the authors of the works [25] stated that the DLC coatings have excellent resistance to corrosion. In this case, the improved corrosion resistance of the DLC layer is another indicator of proper coating quality, and the comparison with the W-DLC was appropriate. However, results show that the progressive type of the W-DLC coating has again superior properties over the conventional process, even when the thickness was less than half of the conventional DLC. This could be beneficial because the application of this coating would result in very small geometrical changes in the bearing components.

## 4. Conclusions

Based on the experimental work carried out to determine the tribological and mechanical properties of the DLC and W-DLC coatings, deposited on 100Cr6 bearing steel, it is possible to state the following conclusions:

- The dry COF, after 500 s of testing, was ≈ 0.137 for the conventional DLC and ≈0.181 for the W-DLC coating. Both coatings have significantly better friction properties when compared to the base material with a dry COF of ≈ 0.5.
- The wet COF after, 500 s of testing, was ≈0.076 for the conventional DLC and ≈0.105 for the W-DLC. In this case, both coatings perform very similarly to the substrate material COF of ≈ 0.1, but this coefficient is starting to increase at the end of the tests, suggesting the start of severe surface wear.
- The combination of wear and the NaCl corrosive environment caused damage to the conventional DLC coating—localities with disrupted layers were observed after the test. No similar damage was observed on the W-DLC coating.
- The W-DLC coating had shown superior corrosion resistance in terms of corrosion potential and corrosion kinetics when compared to the conventional DLC coating.

Based on the obtained experimental results, the progressive type of a tungsten-doped, diamond-like carbon coating has a potential for even better wear and corrosion improvement of bearings, when compared to the conventional DLC in nearshore and offshore applications. The W-DLC coating could prolong the operational lifetime of the bearing while requiring less maintenance.

## Figures and Tables

**Table 1:** Chemical composition of 100Cr6 steel (wt. %).

C	Si	Mn	P	S	Cr	Fe
1.05	0.34	0.42	0.003	0.004	1.48	Bal.

**Table 2:** Results of mechanical properties of the base material and of the coatings (average values).

Parameter	Base Material	DLC Coating	W-DLC Coating
H_IT_ [GPa]	9.9 ± 0.3	11.8 ± 1.8	12.5 ± 1.6
E_IT_ [GPa]	243 ± 7	115 ± 9.0	160 ± 8.1

**Table 3:** Electrochemical properties of the base material and coatings obtained by the PD tests in 3.5% NaCl.

Surface	E_corr_ (mV)	i_corr_ (µA·cm^−2^)	β_a_ (mV/dec)	β_c_ (mVv/dec.)	r_corr_ (µm·Year^−1^)
Base material	−701 ± 11	2.32 ± 0.24	69	220	26.6
DLC coating	−477 ± 8	1.46 ± 0.18	95	173	16.8
W-DLC coating	−172 ± 4	0.11 ± 0.02	323	76	4.7

**Table 4:** Electrochemical properties of the base material and coatings obtained by EIS in 3.5% NaCl.

Surface	R_s_ (Ω·cm^2^)	R_1_ (Ω·cm^2^)	R_2_ (Ω·cm^2^)	R_sum_ (Ω·cm^2^)	CPE_1_ (F·s^n−1^)	CPE_2_ (F·s^n−1^)	n_1_	n_2_
Base material	27 ± 2	2861 ± 62	-	2861 ± 62	3.3 × 10^−4^	-	0.9	-
DLC coating	38 ± 3	5488 ± 84	1292 ± 14	6780 ± 98	3.3 × 10^−6^	2.1 × 10^−5^	0.8	0.8
W-DLC coating	28 ± 3	7416 ± 81	-	7416 ± 81	1.8 × 10^−5^	-	0.8	-

## Data Availability

Not applicable.
